# A Biocompatibility Study of Plasma Nanocoatings onto Cobalt Chromium L605 Alloy for Cardiovascular Stent Applications

**DOI:** 10.3390/ma15175968

**Published:** 2022-08-29

**Authors:** Thithuha Phan, John E. Jones, Meng Chen, Doug K. Bowles, William P. Fay, Qingsong Yu

**Affiliations:** 1 Department of Mechanical Engineering, University of Missouri, Columbia, MO 65211, USA; 2 Nanova, Inc., 1601 S Providence Rd., Columbia, MO 65211, USA; 3 Department of Biomedical Sciences, University of Missouri, Columbia, MO 65211, USA; 4 Department of Medicine, Division of Cardiovascular Medicine, School of Medicine, University of Missouri, Columbia, MO 65212, USA

**Keywords:** plasma nanocoating, NH_3_/O_2_ plasma post-treatment, cobalt chromium L605 alloy, biocompatibility, cardiovascular stent, cell culture, restenosis prevention

## Abstract

The objective of this study was to evaluate the biocompatibility of trimethylsilane (TMS) plasma nanocoatings modified with NH_3_/O_2_ (2:1 molar ratio) plasma post-treatment onto cobalt chromium (CoCr) L605 alloy coupons and stents for cardiovascular stent applications. Biocompatibility of plasma nanocoatings was evaluated by coating adhesion, corrosion behavior, ion releasing, cytotoxicity, and cell proliferation. Surface chemistry and wettability were studied to understand effects of surface properties on biocompatibility. Results show that NH_3_/O_2_ post-treated TMS plasma nanocoatings are hydrophilic with water contact angle of 48.5° and have a typical surface composition of O (39.39 at.%), Si (31.92 at.%), C (24.12 at.%), and N (2.77 at.%). The plasma nanocoatings were conformal to substrate surface topography and had excellent adhesion to the alloy substrates, as assessed by tape test (ASTM D3359), and showed no cracking or peeling off L605 stent surfaces after dilation. The plasma nanocoatings also improve the corrosion resistance of CoCr L605 alloy by increasing corrosion potential and decreasing corrosion rates with no pitting corrosion and no mineral adsorption layer. Ion releasing test revealed that Co, Cr, and Ni ion concentrations were reduced by 64–79%, 67–69%, and 57–72%, respectively, in the plasma-nanocoated L605 samples as compared to uncoated L605 control samples. The plasma nanocoatings showed no sign of cytotoxicity from the test results according to ISO 10993-05 and 10993-12. Seven-day cell culture demonstrated that, in comparison with the uncoated L605 control surfaces, the plasma nanocoating surfaces showed 62 ± 7.3% decrease in porcine coronary artery smooth muscle cells (PCASMCs) density and had comparable density of porcine coronary artery endothelial cells (PCAECs). These results suggest that TMS plasma nanocoatings with NH_3_/O_2_ plasma post-treatment possess the desired biocompatibility for stent applications and support the hypothesis that nanocoated stents could be very effective for in-stent restenosis prevention.

## 1. Introduction

Stents are medical devices that act as scaffolds to open narrowed arteries and restore normal blood flow to the heart. The lack of endothelium and inflammatory responses in vessel walls due to artery damage during stent implantation stimulates smooth muscle cell (SMC) proliferation and migration, leading to in-stent restenosis rates of as high as 20–30% in bare metal stents (BMSs) [1]. First-generation drug-eluting stents (DESs) were developed to reduce restenosis rates by releasing drugs (e.g., paclitaxel and sirolimus) that suppress SMC proliferation. However, stent thrombosis, due to inhibition of re-endothelialization by cytostatic drugs and inflammatory properties of stent polymer coatings, emerged as a key limiting factor of clinical success. Second-generation DESs released newer drugs (e.g., zotarolimus and everolimus) and employed more biocompatible coatings compared to first-generation DESs. Unfortunately, stent thrombosis, potentially occurring even over one year after stent implantation, persisted as a major limitation. To combat the prothrombotic properties of DESs, patients are treated with dual anti-platelet therapy (DAPT) for up to one year after stent implantation. However, DAPT significantly increases bleeding complications compared to single antiplatelet drug therapy [2,3]. While stent technology continues to evolve and improve, the development of new approaches to prevent restenosis, inflammation, and thrombosis remains a high priority, given the high prevalence of coronary artery disease. As an alternative to DES, drug-free modification of the metal surface of stents has considerable potential as a novel approach to improve the biological properties of stents, thereby reducing thrombosis and restenosis rates [4].

Cobalt chromium (CoCr) L605 stents have been widely used because of their excellent mechanical and radiopaque properties and good biocompatibility [5,6]. Thin-strut stent design was found to reduce restenosis and thrombosis events after stent implantation [7]. Despite containing thinner struts compared to stainless-steel stents, CoCr L605 stents have minimal elastic recoil while maintaining X-ray visibility during and after percutaneous coronary intervention [5].

Because of the good compatibility, CoCr surface supports smooth muscle cells (SMCs) triggered by vascular injury [8,9,10]. The proliferation of endothelial cells (ECs) is beneficial for fast and full endothelium coverage, but SMC proliferation and migration through the gaps between stent struts leads to in-stent restenosis [11,12]. Attempts have been made to modify BMS to minimize in-stent restenosis and thrombosis. Choudhary et al. [12] studied EC and SMC adhesion on nanostructured Ti and CoCrMo alloys and found EC and SMC attachment were enhanced, with the ratio of viable EC to SMC slightly increased on nanostructured surfaces. Beschasna et al. [13] investigated titanium oxy-nitride coatings by magnetron sputtering deposition onto 316L stainless-steel stents and found the treated samples showing somewhat better viability of EC compared to 316L stainless-steel surfaces. Shim et al. [14] examined nitrogen ion implantation into CoCr surface using an ion source under plasma environment to prevent platelet adhesion and SMC proliferation. Their results showed that SMC migration and growth decreased to half compared to untreated CoCr counterparts. Their study, however, did not evaluate EC proliferation, which relates directly to endothelium coverage, in-stent restenosis, and thrombosis inhibition.

Surface modification of CoCr stent alloys could change their biological performance, including the cell proliferation and cell adhesion of ECs and SMCs [8,12]. Jones et al. [15] investigated TMS plasma coatings deposited from direct current (DC) and radio frequency (RF) glow discharges onto 316L stainless steel for coronary stent applications. Compared to other coating conditions, EC growth on DC plasma coatings treated with surface modification was significantly enhanced. Osaki et al. [16] used mouse fibroblast cells in an in vitro cytotoxicity test for plasma polymerized tetramethylcyclo-tetrasiloxane coatings on 316 stainless steel. Their results indicated that plasma coatings did not produce a cytotoxicity response to the cells. Wang et al. [17] determined the hemocytolysis rate and the relative growth rate of human umbilical vein endothelial cells (HUVECs) in cytotoxicity experiments of TMS/O_2_ plasma-coated nitinol (NiTi alloy) stents. It was found that the hemocytolysis rate of plasma-coated NiTi stents (0.045%) was significantly lower than that of untreated bare NiTi stents (0.271%). Their results showed that plasma-coated samples provided better biocompatibility than the uncoated nitinol controls.

Low-temperature plasma technique is a common method in thin film deposition and surface modification. Plasma deposition can generate thin film coatings that are highly conformal to substrate surface topography, free of voids, and have excellent adhesion to various substrates. The plasma deposition method is inexpensive and can be achieved with non-toxic gas treatment [4]. Surface chemistries can be changed with plasma modification by incorporating functional groups onto surfaces using appropriate gases or monomers. Stent surfaces are required to be mildly hydrophilic, i.e., water contact angle about 20° to 40° [18], to make them feasible for cell adhesion. However, it is desired that stent surfaces can enhance EC proliferation while preventing SMC proliferation as much as possible. Nitric oxide (NO) can be a candidate to be incorporated into stent surfaces [19]. In vascular systems, ECs are the primary source of NO, which is critical determinant of endothelial health. NO plays pivotal roles in thrombosis prevention and SMC inhibition [14,19]. NO-like functionality-incorporated stent coating would be a very promising approach to meet biocompatibility required for stents but has not been attempted previously. Plasma nanocoatings can be deposited onto CoCr L605 stents, followed by plasma post-treatment with NH_3_ and O_2_, which react to form NO.

Biocompatibility of a plasma coating for stent applications can be evaluated in terms of coating adhesion to metal surfaces, improvement in corrosion resistance, and effects on ECs and SMCs, including proliferation and cytotoxicity. During implantation, a stent needs to withstand high-pressure crimping and expansion processes, which may result in coating cracks and delamination. Metal plastic deformation, mechanical property mismatch between metals and coatings, and poor coating adhesion all can cause cracking and delamination [13]. Corrosion of metal materials reduces mechanical integrity, produces inflammation from metal ion release, and lessens the biomaterial effectiveness. Compared to other common vascular stent materials, such as 316L stainless steel, Ti alloys, and Mg alloys, CoCr L605 alloy is highly corrosion-resistant, even in the electrolytic environment of the human body, due to the development of the passive oxide layer [20,21]. However, the oxide layer of L605 alloys is not passive enough to avoid leakage of metal ions and electrochemical attack, resulting in negative biological effects [22,23]. CoCr-based implants are known to release chromium (Cr) and cobalt (Co) ions [5], which are responsible for allergic reactions. Additionally, the nickel (Ni) content in L605 is about 10%, and its ability to induce allergic reactions and long-term complications, such as restenosis, are well documented [16,23,24].

TMS, known for good reactivity in gas plasmas, is capable of forming a thin film on substrate with stable performance and non-toxicity [25,26]. In this study, trimethylsilyl (TMS) plasma nanocoatings were deposited onto CoCr L605 alloy in DC glow discharge. NH_3_/O_2_ plasma post-treatment was then used to modify the surface chemistry and surface wettability of the TMS plasma nanocoatings to improve their biocompatibility for vascular stent applications.

## 2. Materials and Methods

### 2.1. Material Preparation

CoCr L605 alloy sheets (AMS 5537) purchased from HighTemp Metals (Sylmar, CA, USA) were sectioned into circular coupons (diameter of 15 mm) and square coupons (10 mm × 10 mm). Chemical composition of CoCr L605 substrate includes Co (balance), Cr 20%, W 15%, Ni 10%, Fe 3% (max), Mn 1.5%, Si 0.4% (max), C 0.1%, S 0.03% (max), and P 0.04% (max). The L605 coupons were cleaned with a Detergent 8 solution (Alconox, Inc., White Plains, NY, USA) for 3 h at room temperature in a tumbling jar, rinsed three times with deionized (DI) water by tumbling, rinsed three times with acetone, and finally dried at room temperature with Kimwipes.

CoCr L605 stents (diameter × length = 1.3 mm × 12 mm, Resonetics Israel Ltd., Or Akiva, Israel) were cleaned using the cleaning process recommended by the stent manufacturer. Stents were held in a sample rack immersed in a beaker containing ethanol for 15 min. The beaker containing stents was transferred to an ultrasonic bath held at 50 °C, and sonicated in ethanol for 30 min. Each stent was finally rinsed on the sample rack with fresh ethanol using a squeeze bottle for 3 s. Cleaned stents were air-dried for 15 min before use.

### 2.2. Plasma Nanocoating by DC-Based Glow Discharge

Anhydrous ammonia (purity > 99.99%) was purchased from Airgas (Holts Summit, MO, USA). Oxygen (purity > 99.6%) was purchased from Praxair (Columbia, MO, USA). TMS (purity > 97%) was purchased from Gelest, Inc. (Morrisville, PA, USA). An 80 L bell-jar reactor was used to generate DC glow discharges. Samples were attached to a titanium holder positioned between two titanium electrodes in parallel. The holder acted as the cathode whereas the two square titanium electrodes served as the electrically grounded anodes. The reactor was sealed and evacuated to a base pressure less than 1 mTorr (0.133 Pa) using mechanical and booster pumps connected in series. Gas flow rates were controlled with MKS mass flow controllers (MKS Instruments, Andover, MA, USA) and an MKS 247D readout. The pressure inside the plasma reactor was allowed to stabilize at 50 mTorr (6.67 Pa) for all plasma processing steps using an MKS pressure controller. Pretreatment with oxygen plasma was used to remove organic contaminants on samples surfaces. Plasma pretreatment was conducted with 1 standard cubic centimeter per minute (sccm) oxygen flow at 20 W DC power for 2 min. Following plasma pretreatment, the reactor was pumped to the base pressure of 1 mTorr and then TMS introduced to the reactor at a flow rate of 1 sccm. When TMS pressure stabilized at 50 mTorr (6.67 Pa), plasma was initiated at 5 W DC power for 20 s (coupon samples) and 10 s (stent samples). Plasma post-treatment of the TMS-based nanocoating was carried out with a mixture of NH_3_ and O_2_. NH_3_ and O_2_ flow rates were 2 sccm and 1 sccm, respectively. At pressure stabilization, plasma was initiated at 5 W DC power for 2 min.

### 2.3. Surface Characterization

Plasma nanocoating thickness on L605 coupons was determined by using a microscope-mounted thin-film measurement device (Filmetrics F40-UV, KLA Corporation, Milpitas, CA, USA). The thickness range of measurement the device capable of performing is 4 nm–40 μm. A small sample area is non-destructively analyzed by reflecting light off the nanocoating. The reflectance spectrum is analyzed over a wavelength range (200–2000 nm). The Filmetrics software (FILMeasure, Version 8.12.6 Rev. 0, KLA Corporation, Milpitas, CA, USA) performs curve fitting of the reflectance spectrum to determine the nanocoating thickness. At least three coating thickness measurements were performed on each of three coated samples to determine the mean coating thickness and standard deviation. The plasma nanocoating thickness results were also further confirmed by measuring plasma-nanocoated silicon wafers using the same thin-film measurement device.

Surface contact angles were measured using a contact angle meter/Goniometer-DMe 210 (Kyowa Interface Science Co., Ltd., Eden Prairie, MN, USA) with FAMAS software. Nanocoating stability was assessed by storing the nanocoated coupons in Pyrex dishes at 25 °C for 96 weeks (or 24 months). At least six contact angle measurements were obtained per condition to determine the mean contact angle and standard deviation.

Nanocoating surface chemistry on L605 square coupons was analyzed by X-ray photoelectron spectroscopy (XPS). A Kratos AXIS Ultra DLD X-ray Photoelectron Spectrometer (Kratos Analytical Inc., Chestnut Ridge, NY, USA) utilizing a monochromatic Al Kα X-ray (1486.6 eV) source operating at 150 W was utilized to characterize the coatings to a depth of about 10 nm. The X-ray source take-off angle was set at 90 degrees relative to the coupon surface, and the spot size was 200 μm × 200 μm. The survey scan was performed at 10 mA and 15 kV. Survey spectra were recorded in the binding energy scale from 1200 to −5 eV, and dwell time of 100 ms with two sweeps to resolve peaks. The sample was loaded into the load lock and pumped down to less than 5 × 10^−7^ torr and transferred into the sample analysis chamber. Data were collected at pressures approximately 5 × 10^−9^ torr. Binding energies in the survey and high-resolution spectra were calibrated with respect to C 1 s at 284.6 eV adventitious peak. The acquired data were analyzed by using the Casa XPS software package. Peak fitting was performed using Gaussian deconvolution function. A measured curve (or overlapped peak) was deconvoluted into individual sub-curves by CASA XPS software. These sub-curves (sub-peaks) were assigned as chemical functional groups based on their binding energies. A relative standard deviation parameter of 0.7–1.0 was used for peak fitting as the ideal parameter should be less than 1.2.

### 2.4. Adhesion Tests

Adhesion test of plasma nanocoatings to L605 coupon surfaces was performed by following ASTM D3359. Grid area with six cuts in each direction was scraped onto plasma-nanocoated surfaces, and pressure-sensitive tape was thoroughly adhered over the lattice patterns and then detached. Adhesion was assessed by optical imaging. The adhesion scale was rated from 0B to 5B, as listed in ASTM D3359. The test was repeated three times for each sample.

Adhesion of plasma nanocoatings to CoCr L605 stents was assessed after a crimping and expanding process that mimics stent dilation procedures in clinical practice. Typically, a stent was positioned on a balloon catheter and crimped by Model CX with J-Crimp Station (Blockwise Engineering LLC, Tempe, AZ, USA) to a final crimping diameter of 0.8 mm. The crimped stent was expanded at 12 atm (1.216 MPa) to reach a diameter of 3.2 mm. The stent was then examined under scanning electron microscope (SEM) Quanta 600 FEG equipped with a Schottky Field Emitter (FEI Company, Hillsboro, OR, USA) for coating integrity. An accelerating voltage of 10 kV with probe currents of 4.5 μA was used for the SEM operation.

### 2.5. Electrochemical Characterization

Cyclic polarization (CP) test was performed to assess corrosion rates and pitting susceptibility by following ASTM F2129-15 corrosion protocol. An exposed window area of 0.5 cm × 0.5 cm was created at one end of an L605 strip (0.5 cm × 4 cm) by using epoxy and electric tape to cover the remaining area. A stainless-steel wire covered by electronic tapes was threaded through a borosilicate holder to collect signals from the working electrode (the sample). The sample was placed inside the corrosion cell containing phosphate buffered saline (PBS) (8.0 g/L NaCl, 0.2 g/L KCl, 1.15 g/L Na_2_HPO_4_-7H_2_O, 0.2 g/L KH_2_PO_4_) at body temperature (37 ± 1 °C) in a stirring condition (60 rpm). The exposed area was kept submerged in PBS for 1.5 h for open-circuit measurement prior to CP testing.

CP test was carried out with a PalmSens Emstat (Compact Electrochemical Interfaces- Randhoeve 221, 3995 GA Houten, The Netherlands) using the PSTrace 5.2 software package. Potentials were measured using a Luggin capillary and a saturated calomel electrode (SCE). All potentials given in this work were with respect to the SCE. A conventional 3-electrode arrangement was used with a coupon sample as the working electrode, a SCE as the reference electrode, and a graphite rod of 0.6 cm in diameter as the counter electrode. The electrodes and the nitrogen bubbler were tightly inserted into the cell. Prior to the test, the electrochemical cell was purged with pure nitrogen gas for 30 min, and then purged continuously during the test. The corrosion potential was monitored for 1.5 h in PBS prior to the CP testing, ensuring a stable open circuit potential (OCP). The CP curve was initiated at E_corr_ and scanned in the positive (more noble) direction. The scan was reversed after reaching the vertex potential E_v_ = 0.8 V. The reverse scan was stopped when potentials reached E_corr_.

### 2.6. Immersion Test

An in vitro degradation experiment, i.e., immersion test, was conducted in a simulated body fluid (SBF) solution as described in [27]. SBF (1000 mL) contains NaCl (8.035 g), NaHCO_3_ (0.355 g), KCl (0.225 g), K_2_HPO_4_. 3H_2_O (0.231 g), MgCl_2_.6H_2_O (0.311 g), 1 M HCl (39 mL), CaCl_2_ (0.292 g), Na_2_SO_4_ (0.072 g), and Tris (6.118 g). The SBF was kept at 37 °C and buffered at pH 7.4 exactly. The volume of the electrolyte was calculated based on a 30 mL/cm^2^ volume-to-sample area ratio, according to ASTM-G31-72. During the immersion process, the solution was replaced with fresh SBF every 48 h. After 7 days and 45 days immersion, samples were ultrasonically cleaned in ethanol for 15 min and dried in ambient air. The surface morphology was then examined using SEM. Energy-dispersive X-ray spectroscopy (EDS) was used to determine elemental components on the surface. To determine pitting corrosion, after the immersion test, substrates were cleaned with a 25% chromic acid for 5 min to remove the corrosion products. The samples were then quickly washed with distilled water and dried in ambient air. SEM was used to detect pits. The sample weight was measured before and after immersion by a balance with accuracy of 0.1 mg, and corrosion rate can be obtained based on the weight loss of samples by the following formula:

Corrosion rate = K × W/(A × T × D)(1)

where K = 8.76 × 10^4^ (mm/year), W is mass loss (gram), A is exposure area (cm^2^), T is exposure time (h), and D is density (g/cm^3^) [23].

### 2.7. Ion Releasing Test

Ion releasing test was performed according to ISO 10993-5 and ISO 10993-12. Square L605 samples (10 mm × 10 mm × 0.4 mm) were utilized in this experiment. All edges of samples were polished with sandpaper (grit number of 1000) prior to the test. Extracts were prepared by immersing uncoated and plasma-nanocoated L605 coupons into extract medium (DI water) with surface-area-to-medium ratio of 6 cm^2^/mL at 50 °C for 72 h in a shaking incubator. With distilled water as control, the extracts were measured for various ion concentrations using inductively coupled plasma mass spectrometry (ICP-MS)-NexION 300X (PerkinElmer, Waltham, WA, USA) operated in kinetic energy discrimination mode (KED).

### 2.8. Cytotoxicity Test

International standard ISO 10993-05 and ISO 10993-12 were adopted for cytotoxicity evaluation using porcine artery coronary endothelial cells (PCAECs), which were prepared previously as described [28]. Cell culture medium with FBS (fetal bovine serum, Gibco, Waltham, MA, USA) was used to capture extractable and leachable materials from the test samples. The extracts were tested for cytotoxicity using PCAEC and PCASMC. The cells were cultured in Dulbecco’s Modified Eagle Medium (DMEM, Gibco, USA) supplemented with 10% FBS, 100 U/mL penicillin, and 100 μg/mL streptomycin. Extracts (liquids that result from extraction of the test sample or control) were prepared by immersing uncoated and plasma-nanocoated L605 coupons into culture medium with surface-area-to-medium ratio of 6 cm^2^/mL at 37 °C for 24 h. Extracts of natural latex rubber and high-density polyethylene (HDPE) were used as positive (+) control inhibiting cells and negative (−) control supporting cells, respectively.

Cells (passage number 6) with density of 100,000 cells/well were seeded into 96-well cell culture plates and incubated at 37 °C in a fully humidified air atmosphere containing 5% CO_2_ to allow attachment. After 24 h, media were removed and wells were washed twice with phosphate-buffered saline (PBS). Extracts were added to the 96-well plates with 100 μL/well. The cells were incubated in extracts at 37 °C for 1 day. Cell morphology was examined using an inverted microscope. After the test, extracts and controls were removed from the 96-well plates, and one hundred microliters MTT (3-(4,5-dimethylthiazol-2-yl)-2,5-diphenyl tetrazolium bromide) solution was added to each well and incubated at 37 °C for 4 h. Dimethyl sulfoxide (DMSO) was then added to each well and shaken for 10 min. The spectrophotometric absorbance of the samples was measured by micro-plate reader at the wavelength of 570 nm. Extract medium (blank and non-test samples) without cells was used as control for absorbance reading. Viability reduction compared to the blank was calculated using the following formula:

(2) $$\mathrm{Viability}\;\%=\frac{100\times OD_{570e}}{OD_{570b}}$$

where *OD*_570*e*_ is the density mean value of the 100% extracts of the test sample and *OD*_570*b*_ is the density mean value of the blanks. The lower the metabolic percentage value, the higher the cytotoxicity potential of the test sample. If the viability is lower than 70% of the blank, the test sample is potentially toxic. The usage of ECs instead of L-929 fibroblasts from ISO 10993-05 is described previously [28,29,30,31].

### 2.9. Cell Proliferation

Porcine artery coronary endothelial cells (PCAECs) and porcine coronary artery smooth muscle cells (PCASMCs), which were prepared as described previously [28,32] were resuspended and cultured in 15 mL respective growth media in T-75 flasks. Cells were grown in a humidified atmosphere with 5% CO_2_ at 37 °C from passage 7 to 8. Culture media were replaced every 2 days for both PCASMCs and PCAECs until the cells reached a confluence of 70%. Cells were detached from the culture flasks with trypsin/EDTA (Sigma-Aldrich, St. Louis, MO, USA) and counted with a hemocytometer. PCAEC and PCASMC concentrations were adjusted to 10,000 cells/mL. PCAECs or PCASMCs were seeded onto individual 15 mm diameter coupons of uncoated and plasma-nanocoated L605. The coupons were UV-sterilized for 15 min per side prior to cell culture. Each cell line was cultured with coupons in 24-well cell-culture plates up to 7 days.

After 3-day, 5-day, and 7-day culture, cells were rinsed 2 times with Dulbecco’s phosphate-buffered saline (DPBS), then immersed for 10 min in incubator at 37 °C in 0.25 wt.% Trypsin-EDTA solution diluted with DPBS. Trypsin was neutralized by mixing with media containing FBS and cells were stained by Trypan blue to discriminate viable and non-viable cells. Cell proliferation was determined by a Countess automated cell counter (SPW Industrial, Laguna Hills, CA, USA) at different time points.

Cell morphologies were evaluated by SEM. Cells were rinsed one time with DPBS and fixed by sodium cacodylate (2% glutaraldehyde and 2% paraformaldehyde) for 30 min at room temperature. Then, the cells were dehydrated in a graded series of ethanol (50%, 60%, 70%, 80%, 90%, and 100%) for 10 min each and dried at room temperature in ambient air. The cell attachment and morphologies were examined by an FEI Quanta 600 FEG Environmental SEM (FEI Company, Hillsboro, OR, USA).

### 2.10. Statistical Analysis

The data were expressed as mean ± standard deviation (SD). Each experiment was repeated three times independently, if not indicated otherwise. Data were analyzed using one-way ANOVA followed by the Tukey’s test. A *p*-value of 0.05 or less was considered significant.

## 3. Results

### 3.1. Surface Wettability and Elemental Compositions of the Plasma Nanocoatings

**Figure 1:**
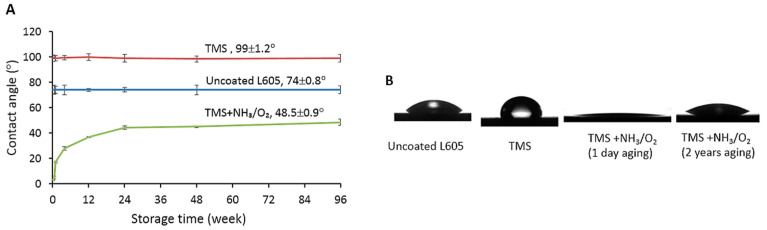
(**A**) Surface static contact angles for uncoated, TMS, and TMS+NH_3_/O_2_ plasma-nanocoated L605 coupons up to 96 weeks, *n* = 6. (**B**) Optical images of spherical water droplets onto uncoated and plasma-nanocoated L605 coupons, *n* = 3. TMS stands for TMS plasma-nanocoated L605, and TMS+NH_3_/O_2_ stands for TMS plasma-nanocoated L605 with NH_3_/O_2_ plasma post-treatment.

The measured thickness of TMS plasma nanocoatings was 20.5 ± 4.0 nm without plasma post-treatment, and 21.5 ± 3.8 nm after NH_3_/O_2_ plasma post-treatment. Figure 1A shows the surface water contact angle results of uncoated and plasma-nanocoated L605 coupons. Uncoated L605 surface had a water contact angle of 74 ± 0.8°. Following deposition of the TMS nanocoating, the contact angle increased to 99 ± 1.2°, indicating hydrophobic surface. However, with NH_3_/O_2_ plasma post-treatment of the TMS plasma nanocoatings, the water contact angle dramatically decreased to 3~5° and then gradually increased to 48.5 ± 0.9° after 96 weeks (24 months) storage in clean and dry air condition. Figure 1B shows the optical images of spherical water droplets (1.2 μL) onto uncoated and plasma-nanocoated L605 coupons. Droplet spread out the NH_3_/O_2_-treated TMS plasma nanocoatings, but it was in almost spherical shape on TMS plasma nanocoating without NH_3_/O_2_ plasma treatment, indicating the hydrophilicity and hydrophobicity on TMS plasma nanocoatings with and without NH_3_/O_2_ modification, respectively.

**Figure 2:**
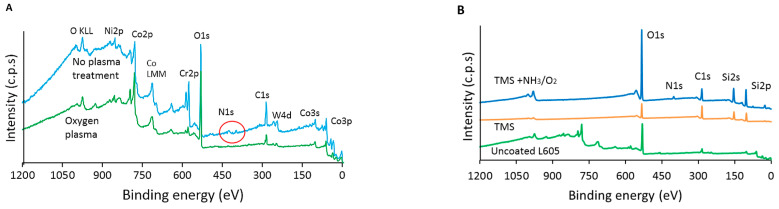

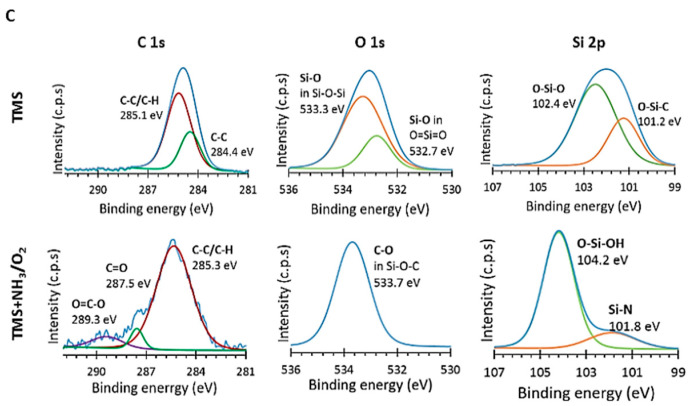
(**A**) XPS survey spectra for uncoated L605 surfaces treated with and without oxygen plasma, *n* = 3. Red circle emphasizes location of N 1s peak. (**B**) XPS survey spectra for uncoated L605, TMS plasma nanocoatings, and TMS+NH_3_/O_2_ plasma nanocoatings, *n* = 3. (**C**). XPS core-level spectra, *n* = 3. TMS stands for TMS plasma-nanocoated L605, and TMS+NH_3_/O_2_ stands for TMS plasma-nanocoated L605 with NH_3_/O_2_ plasma post-treatment.

As shown in Figure 2A and Table 1, XPS survey spectrum for uncoated L605 prior to oxygen plasma treatment indicates the presence of N 1s peak, which disappeared after oxygen plasma pretreatment. After oxygen plasma treatment, that N 1s percentage was reduced from 0.81 ± 0.01 at.% to 0 and C 1s declined from 34.98 ± 1.56 at.% to 25.15 ± 2.46 at.% for L605 samples, implying the clean L605 surfaces prior to the TMS plasma nanocoating process. The contamination removal is considered beneficial to the adhesion of plasma nanocoatings to the L605 substrates. The increase of O 1s percentage from 44.7 ± 2.04 at.% to 61.4 ± 3.12 at.% confirms that the L605 surface not only contains O element inherently in its oxide layer, but also O element incorporated by oxygen plasma treatment.

Figure 2B and Table 2 show a certain amount of carbon (23.33 ± 0.88 at.%) on the L605 alloy surface as characteristic of low contamination with adventitious carbon. In comparison with the uncoated L605, the presence of Si 2p (36.28 ± 1.32 at.%) and the increase of C 1s (from 23.33 ± 0.88 at.% to 47.42 ± 1.6 at.%) indicate that TMS plasma nanocoatings were deposited onto L605 surfaces. On TMS plasma nanocoating surfaces with NH_3_/O_2_ plasma post-treatment (TMS+NH_3_/O_2_), the O 1s amount increases to 39.39 ± 1.06 at.% through NH_3_/O_2_ plasma post-treatment. Conversely, C 1s reduces to 24.12 ± 1.7 at.% due to the etching effect and the surface modification by NH_3_/O_2_ plasma post-treatment in which N, O, and H radicals can react or attach to TMS plasma nanocoating surfaces. TMS plasma nanocoating surfaces with NH_3_/O_2_ plasma post-treatment (TMS+NH_3_/O_2_) have a N 1s concentration of 2.77 ± 0.19 at%, while no trace of N 1s was observed on the surface of uncoated and TMS plasma-nanocoated L605 surfaces.

The XPS core-level spectra using the Gaussian distribution of C 1s, O 1s, and Si 2p in plasma-nanocoated samples are depicted in Figure 2C. The C 1s in TMS plasma nanocoating showed peaks at 284.4 eV and 285.1 eV corresponding to C–C and C–H. Similarly, in TMS+NH_3_/O_2_ plasma nanocoating, the binding energy of 285.3 eV was assigned to C–H. Other peaks at 287.5 eV and 289.3 eV were assigned as C=O and O=C–O polar groups. On TMS+NH_3_/O_2_ plasma nanocoating surfaces, the area percentage C=O and O=C–O polar groups accounted for 6% and 9%, respectively. These polar groups of C=O and O=C–O played important roles in surface hydrophilicity improvement of TMS+NH_3_/O_2_ plasma nanocoatings. XPS core-level O 1s spectrum for TMS plasma nanocoating showed two Si–O peaks, the first one represented Si–O in SiO_2_ at binding energy 532.7 eV and the second bigger peak at 533.3 eV corresponds to Si–O–Si. The O 1s peak in TMS+NH_3_/O_2_ plasma nanocoating is symmetrical and contains no discernable asymmetry or structure. It can be assigned to C–O in Si–O–C or O=C–O. The Si peaks at 101.2 eV and 102.4 eV on TMS plasma nanocoating surface are attributed to the presence of O–Si–C and O–Si–O, respectively. High-resolution Si 2p spectrum for TMS+NH_3_/O_2_ plasma nanocoating presents Si–N at 101.8 eV and O–Si–OH at 104.2 eV. XPS high-resolution spectra confirmed the existence of C–O, C=O, and O–H polar groups on TMS+NH_3_/O_2_ plasma nanocoating surfaces, whereas C–H, C–C, Si–O–Si, and O–Si–O were detected in TMS plasma nanocoating surfaces.

### 3.2. Surface Morphology and Corrosion Resistance of the Plasma Nanocoatings

**Figure 3:**
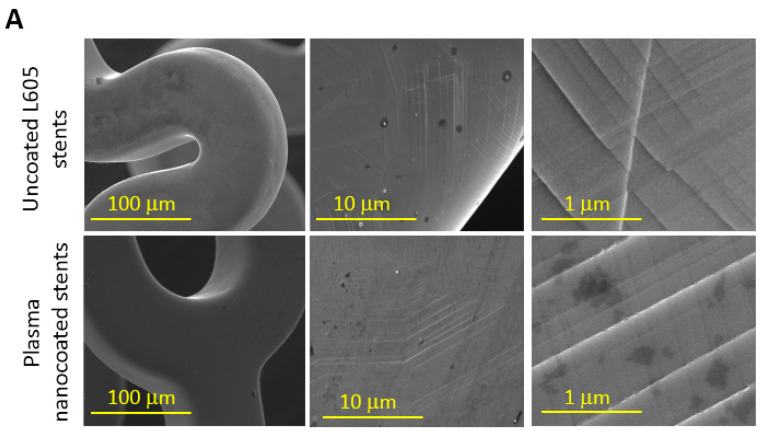

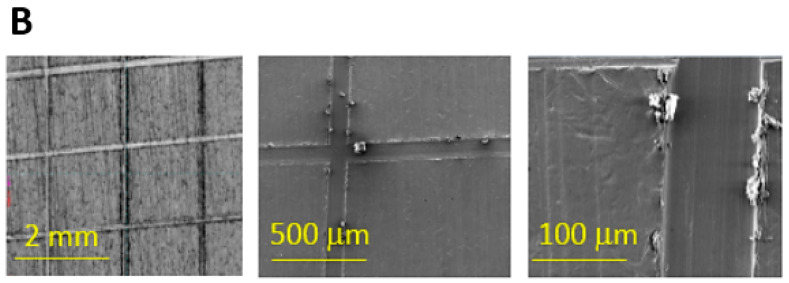
(**A**) SEM images of uncoated L605 stents and plasma-nanocoated L605 stents after crimping and expansion, *n* = 3. (**B**) SEM images of plasma-nanocoated L605 coupons after adhesion test, *n* = 3.

SEM images at high magnification were used to examine any possible failures on stent surfaces such as cracking and coating detachment. Figure 3A shows the images obtained using SEM for uncoated L605 and plasma-nanocoated stents. These images were acquired after stents were crimped down to diameter of 0.75 mm for 30 s and expanded to diameter of 3 mm for 30 s. At low magnification, plasma-nanocoated stent surfaces appeared smoother and more conformal without any defects compared to uncoated L605 stents. At higher magnification, however, many slip lines due to movement of several dislocations were found vertically and horizontally on both stent surfaces after crimping and expanding processes. Neither cracking nor delamination was observed for the plasma nanocoatings. At very high magnification, post-dilated surface texture of uncoated and plasma-nanocoated L605 stents contains multiple shallow and shaft pits and ridges due to dilation processes. No sign of micro-cracking and delamination were observed for plasma-nanocoated stents.

Adhesion was rated by comparing coating pulled-off according to an established standard (ASTM 3359). SEM images of plasma-nanocoated L605 samples after tape test are shown in Figure 3B. Edges of lattice patterns are completely smooth with non-detached layer near the cuts. Residual materials from plasma nanocoating piled up on both sides of grooves are chips produced by plastic material deformation. The adhesion of plasma nanocoating to its substrate was classified as 5B, the highest rating scale for adhesion evaluation with no coating removal. The test was repeated in two other locations on the same sample.

**Figure 4:**
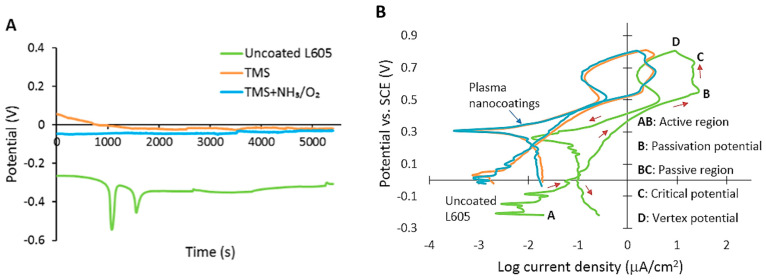

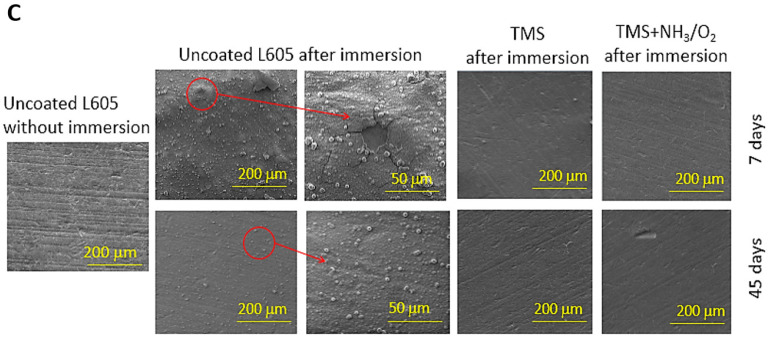
(**A**) Open circuit potential with immersion time for uncoated, TMS, and TMS+NH_3_/O_2_ plasma-nanocoated L605 coupon substrates. (**B**) Cyclic polarization curves for uncoated, TMS, and TMS+NH_3_/O_2_ plasma-nanocoated L605 coupon substrates. (**C**) SEM images for surface morphology of uncoated and plasma-nanocoated L605 coupons after immersion test. TMS stands for TMS plasma-nanocoated L605, and TMS+NH_3_/O_2_ stands for TMS plasma-nanocoated L605 with NH_3_/O_2_ plasma post-treatment.

Open circuit potential (OCP) gives information about stability of the sample surface in a particular corrosion environment. It shows time variation with changes in oxidation tendency of the surface. Corrosion potential (E_corr_) is the electrical potential when no electrical current flows. The OCP curves for uncoated, TMS plasma-nanocoated, and TMS+NH_3_/O_2_ plasma-nanocoated L605 coupons are presented in Figure 4A. Each condition was repeated three times to ensure reproducibility. At the beginning of immersion, E_corr_ of the uncoated L605 dropped abruptly to −0.55 V (vs. SCE). E_corr_ reached an equilibrium value of −0.26 V for uncoated L605 samples within 1.5 h. During the same immersion time, more noble corrosion potentials (approximately −0.03 V) were obtained for TMS+NH_3_/O_2_ plasma-nanocoated L605 surfaces.

Cyclic polarization (CP) curves obtained with uncoated and plasma-nanocoated L605 surfaces are shown in Figure 4B. CP curves show the corrosion current determined by corrosion rates and susceptibility of samples to pitting corrosion. From the CP curves, it was found that plasma-nanocoated L605 samples gave corrosion current of one order of magnitude lower than that for uncoated L605 samples.

Each CP curve consists of a forward scan starting at corrosion potential E_corr_ (point A) up to vertex potential E_v_ (point D), and a reverse scan starting from E_v_ and ending at E_corr_. In CP curves, E_corr_ represents the potential of material in OCP after reaching steady state. In the forward scan, potentials increase with current density in the active region (AB). This is due to the imperfection of substrate surfaces which may contain some defects to activate and begin to propagate corrosion, resulting in an increase in current density. In the passive region (BC), formation of passive layer on substrate surfaces leads to an almost constant current density with the increase of the applied potential. A protective oxide layer was formed in the passive region to prevent metal dissolution from the surface. Followed by the passive region (BC), if current density starts increasing rapidly, point C is termed pitting potential. In Figure 4B, however, the current density starts decreasing with the increase of potentials, implying that no pitting corrosion occurred for uncoated as well as plasma-nanocoated L605 coupons. When potentials reach vertex potential (point D), the reverse scan starts. The intersection of reverse and forward scan was at low potentials of 0.2 V for plasma-nanocoated L605 samples and 0.05 V for uncoated L605.

Figure 4C shows the surface morphology of uncoated and plasma-nanocoated L605 coupons before and after immersion test. There was a layer of adsorption products attached firmly onto the uncoated L605 surfaces. SEM examination of the surface after 7-day immersion showed some cracks and delamination occurring on the adsorption layer. After 45-day immersion in SBF, however, the adsorption layer on uncoated L605 surface seemed to be thicker and more firmly attached without cracking or delamination. EDS data shown in Table 3 indicates the element presence of mostly calcium (Ca) of 35.69 ± 1.25 wt.% and oxygen (O) of 39.06 ± 1.65 followed by phosphorus (P), and a small amount of magnesium (Mg) in the adsorption layer.

To evaluate pitting corrosion, uncoated L605 samples were submerged in chromic acid for 10 min, followed by soaking in DI water and rinsing by acetone. After the mineral adsorption layer was completely removed, no pitting corrosion was observed on uncoated L605 surfaces. However, the corrosion rate was unable to be determined for all samples because the weight loss was out of the detection limit of the laboratory scale used in this study.

### 3.3. Ion Releasing and Cytotoxicity Assessment of Plasma Nanocoatings

**Figure 5:**
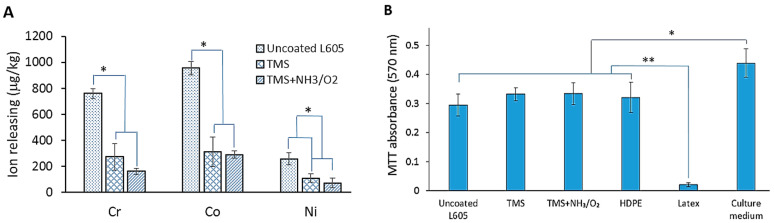

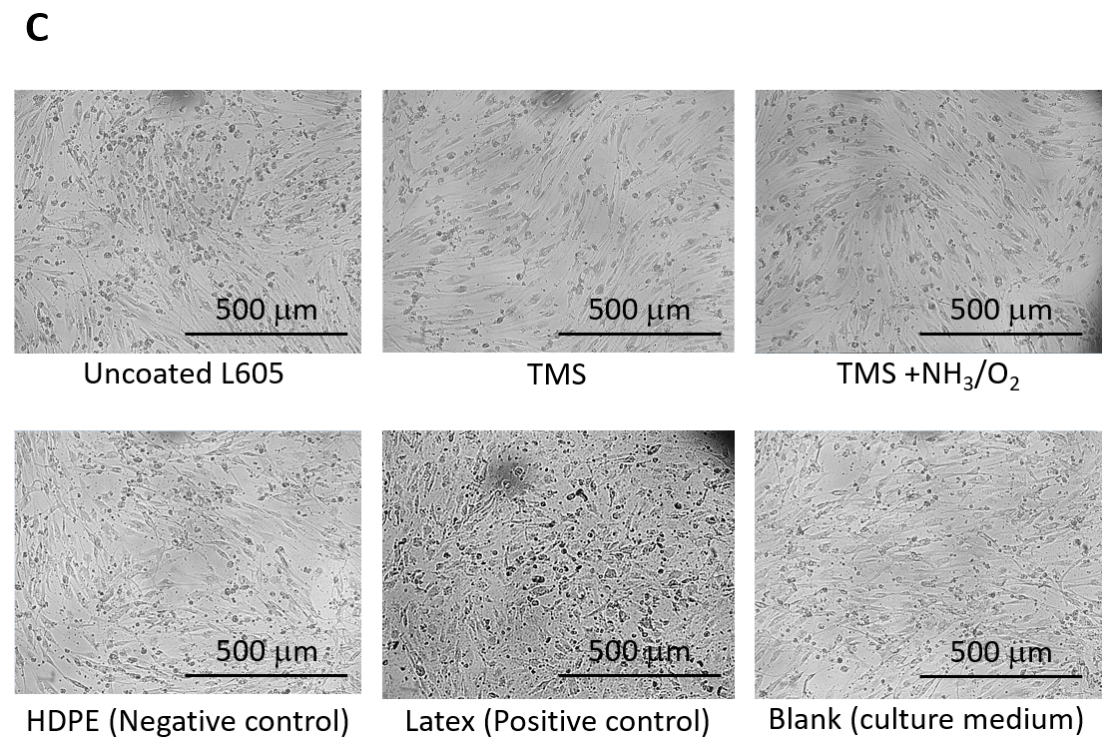
(**A**) Metal ion releasing amount detected from extracts of uncoated, TMS, and TMS+NH_3_/O_2_ plasma-nanocoated L605 coupons. Plotted values are means ± SD (*n* = 3); * *p* < 0.05. (**B**) PCAECs proliferation in cytotoxicity test evaluated by MTT assay. Plotted values are means ± SD (*n* = 6); * *p* < 0.05 and ** *p* < 0.005. (**C**) Optical images (10×) for PCAEC morphology in cytotoxicity test after 3-day incubation, *n* = 3. TMS stands for TMS plasma-nanocoated L605, and TMS+NH_3_/O_2_ stands for TMS plasma-nanocoated L605 with NH_3_/O_2_ plasma post-treatment.

Figure 5A shows metal ion releasing concentration for uncoated L605 and plasma-nanocoated samples into DI water after an ion releasing test. The ion amount detected in uncoated L605 extracts was considerably higher than the plasma-nanocoated L605 samples. The Cr, Co, and Ni ion concentrations in the extracts of TMS plasma nanocoating with and without NH_3_/O_2_ plasma post-treatment modification were similar. In comparison with the uncoated L605 extracts, Cr, Co, and Ni concentrations in extracts of plasma nanocoatings decreased by 64–79%, 67–69%, and 57–72%, respectively, indicating that plasma nanocoatings can reduce ion release from L605 surfaces.

In the cytotoxicity test, sample extracts were used for cell culture to verify if extracts from samples could alter and have negative effect on cell viability. The cytotoxicity results using PCAECs on uncoated L605, TMS, TMS+NH_3_/O_2_, negative control (HDPE), and positive control (rubber latex) are shown in Figure 5B,C. The average absorbance measured from plasma nanocoating extracts is comparable to that collected from negative control (HDPE). Compared to positive control (rubber latex), the average absorbance measured from plasma nanocoating extracts is 10 times higher, suggesting that plasma nanocoating has no negative effect on cell viability. There is no significant difference between MTT absorbance detected from plasma-nanocoated extracts and uncoated L605 extracts. All average viability for uncoated L605, TMS, and TMS+NH_3_/O_2_ plasma nanocoatings is about 70% of viability found in blank (culture medium). Figure 5C presents cells growth after 3 days. While most cells cultured in the extracts of positive controls were dead, there was no obvious difference in the cell growth among PCAECs cultured in plasma nanocoating extracts and negative control extracts, implying no cytotoxicity of plasma nanocoating extract medium to PCAEC proliferation.

### 3.4. Cell Proliferation on Plasma Nanocoatings

**Figure 6:**
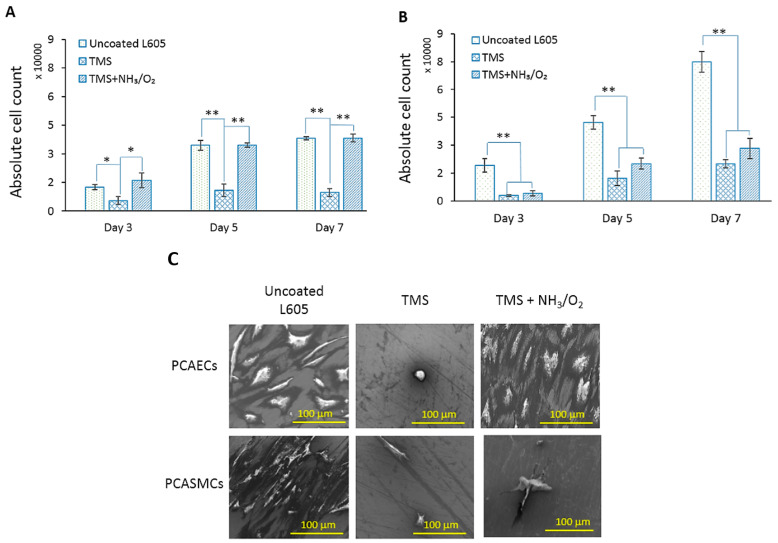
(**A**) Absolute PCAECs counts after 3-, 5-, and 7-day cell culture on uncoated, TMS, and TMS+NH_3_/O_2_ plasma-nanocoated L605 coupons, performed with automated cell counting. Plotted values are means ± SD (*n* = 6); * *p* < 0.05 and ** *p* < 0.005. (**B**) Absolute PCASMCs counts after 3-, 5-, and 7-day cell culture on uncoated, TMS, and TMS+NH_3_/O_2_ plasma-nanocoated L605 coupons, performed with automated cell counting. Plotted values are means ± SD (*n* = 6); * *p* < 0.05 and ** *p* < 0.005. (**C**) SEM images (1000×) for PCAEC and PCASMC cell morphology after 7 days incubation on uncoated, TMS, and TMS+NH_3_/O_2_ plasma-nanocoated L605 surfaces, *n* = 3. TMS stands for TMS plasma-nanocoated L605, and TMS+NH_3_/O_2_ stands for TMS plasma-nanocoated L605 with NH_3_/O_2_ plasma post-treatment.

Figure 6A,B show absolute cell counts for PCAECs and PCASMCs after 3-, 5-, and 7-day incubation. At all three time points, PCAECs counted on uncoated L605 surfaces and TMS+NH_3_/O_2_ plasma-nanocoated L605 surfaces were comparable (*p* > 0.05). Cell adhesion is reduced, indicated by the lower seeding density even at day 3 for TMS plasma nanocoating. Proliferation rate is also lower on TMS (*p* < 0.005), indicating that TMS plasma nanocoating surface is not suitable for PCAEC proliferation. PCASMC cell counts detected on uncoated L605 surfaces increase rapidly at each time point. On the other hand, after day 7, PCASMCs identified on TMS+NH_3_/O_2_ plasma-nanocoated L605 surfaces were reduced about 62 ± 7.3% compared to uncoated L605 samples. Being similar to PCAECs, PCASMC proliferation on TMS plasma-nanocoated L605 surfaces without NH_3_/O_2_ plasma surface modification also showed low growth level. It was also noted that the domination of PCASMCs over PCAECs on uncoated L605 samples increased with time. In contrast, PCAEC levels are significantly higher than PCASMCs at all three sampling time points for TMS+NH_3_/O_2_ plasma-nanocoated surfaces, exhibiting beneficial properties for the selective proliferation of PCAECs over PCASMCs. These results for absolute cell count and cell growth rates confirmed that TMS+NH_3_/O_2_ plasma nanocoatings have the capability to simultaneously promote PCAEC growth and prevent PCASMC proliferation.

Figure 6C shows the cell morphologies of PCAECs and PCASMCs examined with SEM images at high magnification (1000×). PCAECs looked healthy on both uncoated and TMS+NH_3_/O_2_ plasma nanocoating L605 surfaces, with cells spreading and flattening in multi-angular shapes showing visible filopodia to form bridges among cells. PCASMCs on uncoated L605 surfaces showed robust state with spreading and directional cells, implying that PCASMCs were migrating along the bare metal. In contrast, a few PCASMCs were found on TMS+NH_3_/O_2_ plasma-nanocoated L605 surfaces, with cells being mostly rounded and showing poor migration. PCAECs and PCASMCs detected on TMS plasma nanocoating without plasma post-treatment showed smaller and round shape, indicating that TMS plasma nanocoatings are not favorable surfaces for both PCAECs and PCASMCs.

## 4. Discussion

Biocompatibility of stent surfaces relates to surface chemistry, excellent adhesion, less ion releasing, no potential cytotoxicity, good PCAEC proliferation, and less PCASMC viability. TMS plasma nanocoatings are hydrophobic due to the existence of C–C and C–H [33], Si–O, and O–Si–C and O–Si–O [34,35] shown in high-resolution spectra C 1s, O 1s, and Si 2p, respectively (Figure 2C). The very hydrophilic surfaces of TMS plasma nanocoatings with NH_3_/O_2_ plasma post-treatment are attributed to the polar groups C=O and O=C–O [36] in C 1s core-level spectrum, C–O in Si–O–C [33,37,38,39] or O=C–O [40] in O 1s core-level spectrum, and O–Si–OH at 104.2 eV [35] in Si 2p core-level spectrum. The peak position shift to 104.2 eV can be attributed to the siloxane unit with a hydroxyl group substituted for a methyl group. Hydrophobic recovery occurs due to thermal motion and rotation of polar groups attached to the plasma nanocoating surfaces. Chemical groups or chain segments at plasma nanocoating surfaces have greater freedom than those in the bulk and tend to rearrange to equilibrate with ambient environments. These rearrangements directly affect the hydrophilicity stability of the plasma nanocoatings and subsequent water contact angle stabilization over the aging period. Hydrophilic stability after 6 months can be explained by the formation of cross-linkage in the plasma nanocoatings that suppress the rearrangement of the polar group on the surface [40,41,42,43]. All the nanocoated samples utilized in this study were aged for three months prior to any surface characterization experiments and bio-related testing. As shown in Figure 2B and Table 2, nitrogen element (N) was incorporated into TMS plasma nanocoating surfaces by NH_3_/O_2_ plasma post-treatment. The ratio of O/Si increases almost three times in TMS+NH_3_/O_2_ compared to that for TMS plasma nanocoating, while the ratio of C/Si decreases by half. These results indicate that NH_3_/O_2_ plasma post-treatment introduced N and O elements onto TMS plasma nanocoating surfaces to form various N- and O-containing polar groups and thus result in more hydrophilic surfaces.

Adhesion is highly important for the practical application of coatings. The adhesion of plasma nanocoatings may be improved by surface contamination removal on L605 surfaces by oxygen plasma pretreatment. As shown by XPS results presented in Figure 2A and Table 1, after oxygen plasma pretreatment, N 1s percentage became zero and the C 1s percentage was significantly reduced on L605 coupon surfaces, implying effective cleaning of contamination on L605 surfaces. In plasma deposition, adhesion of plasma nanocoatings to substrates is attributed to Si–O–Co and Si–O–Cr bond formation between TMS plasma nanocoatings to the surface oxide layer of uncoated L605. A very thin coating layer (~20 nm) which minimizes internal stresses occurring in plasma nanocoatings is another reason that plasma-nanocoated stents did not undergo cracking and delamination during the dilation process. Mechanical stability testing evaluates coating defects such as cracking or peeling off from the substrate surfaces. Stent coatings are susceptible to large strains during crimping in manufacturing process and expansion in a clinical implantation procedure. To implant stents into coronary arteries, a stent is crimped onto a balloon catheter. Along with a guide catheter and a guide wire, the stent mounted on the balloon catheter is tracked to the lesion area and expanded to open the artery and restore blood flow. The evaluation of the plasma nanocoating on stents following plastic compression and expansion is necessary.

NH_3_/O_2_-modified TMS plasma nanocoatings show corrosion improvement compared to uncoated L605 by enhancing corrosion potential E_corr_, reducing corrosion current density, and exhibiting no adsorption mineral products. The E_corr_ drop shown in OCP plot for uncoated L605 (Figure 4A) was due to PBS solution reached the substrate by diffusion and penetrated through the surface oxide layer on the exposed L605 surface [44]. As the immersion time increased, more oxides and corrosion products were formed to result in a more noble E_corr_ [23,24]. The more noble E_corr_ in the OCP plot indicates an improved anodic protection as compared with the uncoated counterpart. The OCP measurements for plasma nanocoatings show a steady evolution during immersion time, pointing to stronger surface passivation and homogeneous and void-free nanocoatings [20,23]. The lower magnitude of corrosion current in Figure 4B implies that plasma-nanocoated surfaces offer stronger corrosion protection due to lower corrosion rates than that of the uncoated L605. The reverse scan came above the forward scan, indicating that materials are resistant to pitting and crevice corrosion [45]. In other words, the existence of negative hysteresis loop (counterclockwise loop) points out that localized corrosion did not occur for those materials. In the absence of pitting, the reverse scan failed to intersect the forward scan to identify re-passivation potential. The interaction of reverse and forward scans was not considered as re-passivation potential because under these low intersection values reverse current density was higher than forward current density. The CP corrosion test data verify that both uncoated and plasma-nanocoated L605 showed no pitting corrosion, confirming high pitting corrosion resistance of uncoated L605, as reported in the literature [21,22]. The high pitting corrosion resistance was expected by the topmost oxide layer of cobalt oxide followed by chromium oxide (Cr_2_O_3_) [20]. Plasma-nanocoated L605 coupons contain Si–O bonding, as indicated in Figure 2C, which provided good corrosion resistance because of the lower dissolution rate of Si–O bonding compared to other metal oxides [46,47]. Additionally, the presence of Si (Figure 2B,C) can account for an improved anodic protection of plasma nanocoatings on L605 surfaces, shown by OCP curves in Figure 4A. Si–O also can be the reason for less ion releasing from plasma-nanocoated samples compared to uncoated L605, as shown in Figure 5A. The higher levels of Co and Cr ion releasing in all samples compared to Ni can be attributed to Co and Cr oxides that form on the top surface of uncoated L605 [48]. Ni is not commonly found on the very top surface of uncoated L605, but Ni can be exposed at the cutting surfaces of samples. It is known that Ca is a large component of plaque which blocks the blood flow through stents, so the Ca-containing mineral layer may need further assessment [44,45,46,47,48,49]. Plasma-nanocoated samples showed no sign of corrosion after ultrasonically cleaning with acetone, as shown in Figure 4C.

Cytotoxicity is one of the most basic tests of biocompatibility of stent materials. According to ISO 10993-5, if viability is reduced to less than 70% of the blank (culture medium only), it is a sign of cytotoxicity. In Figure 5B,C, PCAEC viability levels of uncoated and plasma nanocoated L605 extracts show higher than 70% of the viability performed in blank (culture medium), indicating no cytotoxic potential for uncoated and plasma-nanocoated L605 samples. The negative control HDPE extracts have similar viability to uncoated L605, TMS plasma nanocoating, and NH_3_/O_2_-modified TMS plasma nanocoating, suggesting that maybe PCAECs are not as hardy as the L-929 fibroblasts mentioned in the ISO 10993. This indicates that either uncoated or plasma-nanocoated L605 samples did not have significant impact on PCAECs cell viability, thus plasma nanocoatings could be considered as nontoxic and biocompatible materials.

Cell attachment and proliferation were influenced by surface wettability change. On TMS plasma nanocoating surfaces, PCAEC and PCASMC attachment was prevented due to the hydrophobic surfaces. The reason can be the protein interface between cells and substrates. If the surface is hydrophobic, adsorbed proteins will denature [14,44]. In Figure 6C, PCAECs and PCASMCs failed to spread on TMS plasma nanocoating surfaces which are hydrophobic with a contact angle of 99°. However, if the surface is too hydrophilic, proteins fail to attach, resulting in poor cell adhesion. Protein attachment is ideal on moderately hydrophilic surfaces, leading to cell spreading and binding on the substrates [14]. Contact angles of 74° and 48.5° for uncoated and TMS+NH_3_/O_2_ plasma-nanocoated L605 surfaces, respectively, are considered favorable for cell growth. In Figure 6C, PCAECs appear robust and spread-out on uncoated and TMS+NH_3_/O_2_ plasma-nanocoated L605 surfaces. Compared to spreading cells, elongated cells are considered more proliferative and prone to migration [44]. PCAEC morphology on TMS+NH_3_/O_2_ plasma nanocoating surfaces appears more elongated and directional compared to PCAECs detected on uncoated L605 surfaces, potentially resulting in higher PCAEC proliferation and migration on TMS+NH_3_/O_2_ plasma-nanocoated L605 surfaces.

Surface wettability is not the only factor influencing cell growth. Unlike the similarity of PCAEC proliferation on uncoated and TMS+NH_3_/O_2_ plasma-nanocoated L605 surface, PCASMCs were inhibited by TMS+NH_3_/O_2_ plasma nanocoatings but supported by uncoated L605, as shown in Figure 6A,B. NO may be attributed to that phenomenon because NO plays a crucial role in inhibition of SMC proliferation and adhesion. As shown in Figure 2B and Table 2, N and O elements were incorporated into surface chemistry of TMS plasma nanocoatings through NH_3_/O_2_ post-treatment. These N and O elements can be present in the form of various N- and O-containing chemical groups attached to the plasma nanocoating surfaces. There are some mechanisms for NO inhibitory effect on SMCs, adhesion, and proliferations, including inhibition of collagen production and DNA synthesis [49,50,51]. The in vitro results obtained in this study indicated that TMS+NH_3_/O_2_ plasma nanocoatings had similar effects on inhibiting PCASMCs.

## 5. Conclusions

CoCr L605 is known as a biocompatible material due to its cell-supporting surface, good corrosion resistance, and low toxicity potential. However, L605 stents have limitations in in-stent restenosis and thrombosis. In this study, TMS plasma nanocoatings with NH_3_/O_2_ plasma post-treatment/surface modification were deposited onto CoCr L605 surfaces to improve their biocompatibility for stent applications. Excellent adhesion, good corrosion behavior, low ion releasing, and non-toxic potential of the plasma nanocoatings post-treated with NH_3_/O_2_ plasma are expected to have the capability to minimize inflammation and thrombosis events. The moderate surface hydrophilicity from NH_3_/O_2_ plasma post-treatment retained endothelial cells on the plasma nanocoating surfaces as comparable to those of uncoated L605. In contrast, smooth muscle cell growth decreased about 62 ± 7.3% on plasma-nanocoated surfaces, as compared with that on the uncoated L605 controls. The N- and O-containing surface chemistry on the plasma nanocoatings is attributed to prevention of smooth muscle cell proliferation, which is a primary cause of restenosis. Therefore, TMS+NH_3_/O_2_ plasma nanocoating could be a promising candidate for minimizing both restenosis and thrombosis in stent applications.

## Figures and Tables

**Table 1:** XPS surface atomic percentages (at.%) of uncoated L605 before and after oxygen (O_2_) plasma treatment, *n* = 3.

Elements	O 1s	C 1s	N 1s	Co 2p	Cr 2p	Ni 2p	W 4d
Uncoated L605	44.70 ± 2.04	34.98 ± 1.56	0.81 ± 0.01	7.1 ± 0.05	10.01 ± 1.12	0.74 ± 0.00	1.21 ± 0.01
O_2_ plasma-treated L605	61.40 ± 3.12	25.15 ± 2.46	0	9.91 ± 1.27	1.58 ± 0.03	1.48 ± 0.01	0.48 ± 0.00

**Table 2:** XPS surface atomic percentages (at.%) of uncoated L605 and plasma-nanocoated coupons, *n* = 3.

Elements	O 1s	N 1s	C 1s	Si 2p	O/Si	N/Si	C/Si
Uncoated L605	50.03 ± 2.02	0	23.33 ± 0.88	0	N/A	N/A	N/A
TMS	16.31 ± 0.28	0	47.42 ± 1.60	36.28 ± 1.32	0.45	0	1.31
TMS+NH_3_/O_2_	39.39 ± 1.06	2.77 ± 0.19	24.12 ± 1.70	31.92 ± 1.08	1.23	0.086	0.76

**Table 3:** EDS data of weight percentages (wt.%) of the adsorbed products on uncoated and plasma-nanocoated coupon surfaces after immersion test, *n* = 3. N/D: None detected.

Elements	Uncoated L605 (wt.%)	Plasma Nanocoatings (wt.%)
Ca	35.69 ± 1.25	N/D
P	19.54 ± 1.79	N/D
Mg	1.08 ± 0.37	N/D
O	39.06 ± 1.65	2.65 ± 0.03

## Data Availability

Not applicable.
